# Isolation and preservation of schistosome eggs and larvae in RNA*later*^® ^facilitates genetic profiling of individuals

**DOI:** 10.1186/1756-3305-2-50

**Published:** 2009-10-23

**Authors:** Bonnie L Webster

**Affiliations:** 1 Biomedical Parasitology, Wolfson Wellcome Laboratories, Department of Zoology, The Natural History Museum, Cromwell Road, London SW7 5BD, UK

## Abstract

Although field-sampling procedures to capture gDNA from individual schistosome larval stages directly from their natural hosts exist, they do pose some technical and logistical challenges hampering certain epidemiological studies. The aim of this study was to develop, refine and evaluate an alternative methodology, which enables better preservation of large numbers of individual schistosome larval stages and eggs collected in low resource endemic areas, to provide PCR-quality DNA for multi-locus genetic analysis. The techniques reported here present simple and effective short-term field and long-term laboratory preservation and storage systems for individually sampled schistosome eggs and larval stages using a commercially available aqueous stabilisation reagent, RNA*later*^® ^eliminating the need for more cumbersome resources such as refrigerators, heaters and centrifuge equipment for immediate specimen processing. Adaptations to a general gDNA extraction method are described, that enables the acquisition of a gDNA extract (~50 μl), facilitating multiple molecular analyses of each sampled schistosome. The methodology provided PCR-quality mitochondrial and nuclear DNA from laboratory cercariae, miracidia and eggs that had been stored at up to 37°C for 2 weeks and at 4°C for 6 months and also from field collected samples. This present protocol provides significant epidemiological, ethical and practical advantages over existing sampling methods and has the potential to be transferred to studies on other organisms, especially where specimens are unable to be seen by the naked eye, are difficult to handle and need to be obtained from a field environment.

## Findings

Advances in molecular approaches to investigate schistosome biology have added further dimensions to aspects of monitoring and surveillance of schistosomiasis. Molecular epidemiological studies have enabled pioneering investigations into many important topics possibly associated with the population genetics of these parasites [1], but studies can be hampered by the inaccessibility of adult schistosomes, which reside within the blood vascular system of the mammalian host. Improvements in sampling procedures have enabled the direct sampling of individual larval stages in field situations enabling researchers to avoid the problems associated with laboratory passage [2]. However, these procedures do have some limitations such as; the need for immediate cooling and processing [3] and single PCR analysis [2].

The aim of this study was to develop and refine better methodologies for short-term tropical field and long-term laboratory preservation of large numbers of individual larval schistosomes and eggs sampled directly from natural hosts, in order to obtain gDNA suitable for multiple molecular analyses on each individual specimen. The protocol will facilitate more detailed and ethically advantageous sampling of schistosome populations for genetic profiling.

**Figure 1:**
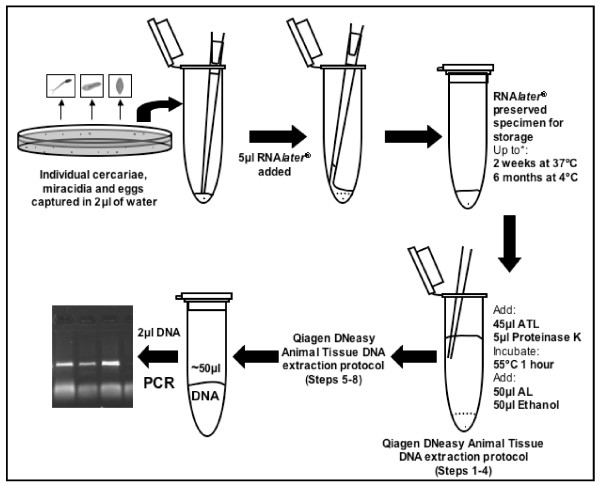
**Schematic of the RNA*later*^® ^preservation and gDNA extraction of individual schistosome larval stages and eggs**. ATL and AL are lysis buffers supplied in the Qiagen DNeasy Blood and Tissue Kit. *These were the longest times and highest temperatures tested but it is expected that the samples can be preserved for much longer.

*Schistosoma haematobium *cercariae and miracidia were obtained from laboratory maintained isolates as described by Webster *et al*., 2009 [4]. Eggs were also collected prior to hatching. Samples were collected using a pipette and washed, to avoid contamination, by transfer to a new Petri dish containing bottled spring water. Individual larval stages or eggs were then captured in 2 μl of the water using a Gilson pipette and ejected into the bottom of a 0.2 ml Eppendorf tube. Each tube was checked for the presence of the individual larval stage by visualization under a binocular microscope and then 5 μl of RNA*later*^® ^was added to preserve the sample and the tube was sealed (Fig. 1). Due to difficulties in catching free swimming larval stages in just 2 μl of water some of the samples were caught in 4 μl of water and preserved by adding 10 μl of RNA*later*^®^. As part of a larger field study in Senegal, schistosome miracidia were hatched directly from eggs recovered from human urine and faecal samples as described in Huyse *et al*., 2009 and preserved as above [5]. Ethical approval was obtained under the EU CONTRAST programme. All samples were subjected to the conditions as described in Table 1.

**Table 1:** Exposure conditions of the preserved samples and resulting PCRs produced from their gDNA extracts

**Sample sets**	**Conditions**		**PCR result**	
**Lab**.	**Temperature°C**	**Time**	**COX1**	**ITS**

1	26-28	3 days	✓	✓

2	26-28	1 week	✓	✓

3	26-28	2 weeks	✓	✓

4	37	3 days	✓	✓

5	37	1 week	✓	✓

6	37	2 weeks	✓	✓

7	4	6 months	✓	✓

8	-20	6 months	✓	✓

**Field**	**Conditions**		**COX1**	**ITS**

9	Tropical field (28-32°C) setting for 2 weeks + transport by plane as hold luggage		✓	✓

10	As in 9 and then storage at 4°C for up to 6 months		✓	✓

11	As in 9 and then storage at -20°C for up to 6 months		✓	✓

Each sample set 1-8 included 2 of each larval stage (cercariae, miracidia) and eggs preserved in 5 μl or 10 μl of RNA*later*^® ^solution. Sample sets 9-10 contain 2 miracidia collected in the field and stored in 5 μl RNA*later*^®^. ✓ corresponds to 100% of the samples producing a positive PCR and sequences of good quality.

In the laboratory gDNA from each individual was extracted using the DNeasy Tissue kit (Qiagen). The samples were briefly centrifuged to re-collect them at the bottom of the Eppendorf tube and then the DNeasy animal tissue extraction protocol was followed with the following modifications. One quarter volumes of the reagents were used for the digestion and DNA precipitation steps 1-4 of the manufacturers protocol and the reagents were added directly to the tubes containing the RNA*later*^® ^preserved samples followed by incubation for just 1 h (Fig 1). In the final step the gDNA was eluted in a total of 50 μl. The concentration of the gDNA extracted was determined using a NanoDrop^® ^ND-1000 spectrophotometer.

**Figure 2:**
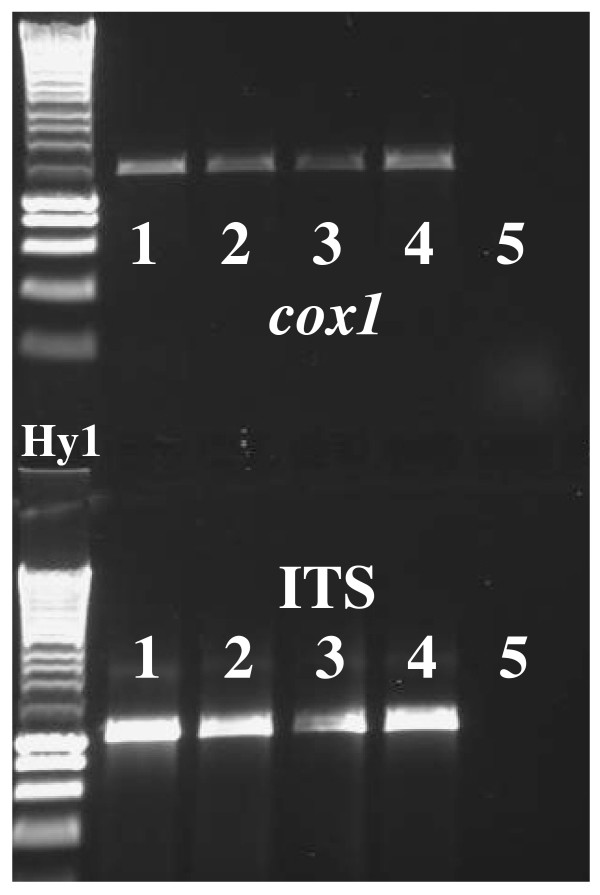
**Gel image showing 3 μl of PCR amplicons amplified from DNA extracts of individual schistosome larval stages and eggs, preserved in RNA*later*^®^**. The image was captured using the UVP gel doc system. **Tracks **(Hy1 = Hyperladder 1) 1. RNA*later*^® ^preserved adult worm (Positive control). 2. RNA*later*^® ^preserved miracidia. 3. RNA*later*^® ^preserved cercaria. 4. RNA*later*^® ^preserved egg. 5. (Negative control) 2 μl of the water that the miracidia were hatched in was mixed with 5 μl of RNA*later*^®^. The DNA extraction protocol was carried out on this mix and the resulting elute used in the PCR.

The complete nuclear ITS rDNA and partial *cox1 *mtDNA were amplified separately for each individual schistosome. PCR amplifications were performed using 2 μl of each gDNA extract as described in Table 2. Positive reactions were identified by agarose gel electrophoresis (Figure 2). To check the quality of the PCR products *cox1 *and ITS amplicons from a single egg, cercaria and miracidium from each of the sample sets (Table 1) were purified using the Qiagen PCR purification Kit (Qiagen) according to the manufacturer's protocol and sequenced using the original PCR primers. The sequences were edited using Sequencher (GeneCodes Corp.) and identified by BLAST. The quality of the PCR products was ascertained by visualisation of the raw sequence chromatograms.

**Table 2:** PCR primers and conditions

	**Primers**	
	
**DNA region**	**Forward**	**Reverse**
COX1	Schisto5'	Schisto3'
	TCTTTRGATCATAAGCG	TAATGCATMGGAAAAAAACA

ITS	ITSF TAACAAGGTTTCCGTAGGTGAA	ITSR TGCTTAAGTTCAGCGGGT

25 μl PCR reactions were carried out using Ready-to-go PCR Beads (Amersham) and 10 pmol of each primer for the DNA region to be amplified as above. Thermal cycling conditions were: 2 min denaturing at 95°C: 40 cycles of 30 s at 95°C, 30 s at 40°C, 2 min at 72°C; followed by final extension period of 7 min at 72°C. Positive amplification was checked by 0.8% Gel Red agarose gel electrophoresis of 3 μl of each amplicon, which were visualised using a UVP gel doc system (Fig 1).

This methodology enabled the capture and preservation of individual larval stages for molecular epidemiological studies in very basic settings. PCR-quality gDNA was obtained from samples caught in small amounts of water and preserved by adding 2.5× the volume of RNA*later*^®^, half the recommended amount by the manufacturer proving highly cost effective.

Free-swimming larvae can be difficult to catch and are often unknowingly not captured or stick to the side of the pipette tip. An advantage of this preservation technique over existing methods [2] is that, the presence of a single larval stage in the tube can be checked prior to preservation. This eliminates DNA contamination from multiple individuals being caught together and also PCR failures due to capture failures, saving time and resources.

The results show that the DNeasy extraction method used was not significantly affected by the presence of the water or RNA*later*^® ^allowing the digestion buffers to be added straight to the sample. Thus excluding any need for relocating, drying or re-hydrating, which is time consuming and can lead to damage and or loss of the sample. This method proved equally good for cercariae, miracidia and eggs. Storage of eggs can be valuable when there are time constraints or hatching of miracidia is proving difficult, moreover egg DNA is not always liberated when stored on Whatman FTA^® ^cards.

PCR-quality gDNA was obtained from RNA*later*^® ^preserved samples from all time lines and temperatures tested. The results show that the preserved samples were stable at temperatures up to 37°C for up to 2 weeks. This was thought to be the maximum temperature and duration that the samples would be expected to endure following field collection before a refrigeration source was available. The samples also proved stable for up to 6 months at both 4°C and -20°C. It is predicted that the samples can remain stable for many more months, especially at -20°C as suggested in the RNA*later*^® ^protocol. This long-term preservation capability together with the fact that the individual samples can be stored in a 96-well plate format, requiring less space, provides options for long-term storage solutions. This also eliminates the immediate need to process the material, allowing greater sample sizes to be collected if required.

Positive *cox1 *and ITS PCRs were achieved from gDNA extracted from each individually RNA*later*^® ^preserved specimen (Table 1) even though the concentrations of gDNA were low (1.6-3.65 ng/μl for eggs/miracidia and 3.1-4.5 ng/μl for cercariae). The sequences obtained from selected amplicons corresponded to the correct organism and gene region being genotyped and the sequences were of high quality.

From each individual larval stage enough gDNA (48-50 μl) was obtained to carry out 24-25 separate PCRs enabling multiple-locus genetic analyses of the same individual and also repeat PCRs if necessary. This overcomes one major restriction of the Whatman FTA^® ^card methodology [2] where only a single PCR can be routinely carried out on each individual. As both nuclear and mitochondrial DNA regions have been amplified the preservation method does not show any disadvantages/advantages in preservation of the different kinds of DNA. Although only the *cox1 *and ITS DNA regions were amplified no reasons exist for any limitations as to the type of population genetic analyses that could be done utilizing other DNA regions, such as microsatellites, 'barcoding', Snapshot™ and RAPD's [1]. Another added advantage is the preservation of RNA facilitating gene expression studies, however the material would need to be treated as stated by the manufacturers recommendations to ensure RNA stabilisation.

The DNeasy extraction method proved highly efficient and effective, however it is expected that other DNA extraction methods would be suitable. The scaling down of the DNeasy reagents is cost effective for processing large populations and high throughput DNeasy 96 Plates can be used making the process more efficient, especially if specimens are originally preserved in 96-well plates.

This preservation method proved just as robust for the laboratory and field preserved material; the field trial was considered typical field-based collecting conditions. This technique will be of particular value for future biological fieldwork, especially where there is no immediate access to cold storage or equipment for immediate processing of the material. Introduction of this sampling and preservation technique may open up geographical areas that were previously excluded due to practical difficulties. Another important practical benefit is that RNA*later*^® ^is innocuous and is classified under the IATA-DGR as not dangerous goods, facilitating easier transportation by air.

To conclude, the methods and protocols described here for the collection, preservation and DNA extraction of schistosome larval stages and eggs using readily available reagents and kits are simple, robust, efficient, reliable, practical, cost effective and have proved extremely useful in a basic field laboratory. In line with Whatman FTA^®^ cards this approach enables parasite genetic analysis without the use of laboratory animals and is in keeping with the '3Rs', the foundation of animal experimentation ethics [6]. Furthermore this methodology allows multi-locus genotypic analysis of schistosome larval stages directly from the field environment. The development and refinement of such sampling techniques is extremely important as studies have shown that examining larval samples directly from their naturally infected hosts offers epidemiological, ethical and practical advantages [1,2]. This is particularly topical due to the recent findings such as the surprising amount of genetic variation present within schistosome populations [7], the discovery and identification of new *Schistosoma *species [8] and interactions between species [5,9]. Future studies warrant larger sample sizes of individuals and multi-locus approaches for precise identification and genetic analysis.

## Abbreviations

gDNA: Genomic DNA; mtDNA: mitochondrial DNA; ITS: intergenic spacer; rDNA: ribosomal DNA; *cox1*: cytochrome oxidase subunit 1; BLAST: Basic Local Alignment Search Tool; IATA-DGR: International Air Transport Association: Dangerous Goods Regulations; 3R's: reduce, reuse and recycle

## Competing interests

The author declares that they have no competing interests.

## Authors' contributions

BW conceived the study, carried out the practical, laboratory, field and molecular work and also wrote the manuscript.

## References

[B1] Rollinson D, Webster JP, Webster BL, Nyakaana S, Stothard JR (2009). Genetic diversity of schistosomes and snails: significance for control. Parasitology.

[B2] Gower CM, Shrivastava J, Lamberton PHL, Rollinson D, Webster BL, Emery A, Kabatereine NB, Webster JP (2007). Development and application of an ethically and epidemiologically advantageous assay for the multi-locus microsatellite analysis of *Schistosoma mansoni*. Parasitology.

[B3] Agola LE, Steinauer ML, Mburu DN, Mungai BN, Mwangi IN, Magoma GN, Loker ES, Mkoji GM (2009). Genetic diversity and population structure of *Schistosoma mansoni *within human infrapopulations in Mwea, central Kenya assessed by microsatellite markers. Acta Tropica.

[B4] Webster BL, Rollinson D, Stothard JR, Huyse T (2009). Rapid diagnostic multiplex PCR (RDPCR) to discriminate *Schistosoma haematobium *and *S. bovis*. Journal of Helminthology.

[B5] Huyse T, Webster BL, Geldof S, Stothard JR, Diaw OT, Polman K, Rollinson D (2009). Bidirectional introgressive hybridization between a cattle and human schistosome species. PLoS Pathogens.

[B6] Wolfensohn S, Lloyd M (1999). Handbook of Laboratory Animal Management and Welfare.

[B7] Stothard JR, Webster BL, Weber T, Nyakaana S, Webster JP, Kazibwe F, Kabatereine NB, Rollinson D (2009). Molecular epidemiology of *Schistosoma mansoni *in Uganda: DNA barcoding reveals substantive genetic diversity within Lake Albert and Victoria populations. Parasitology.

[B8] Hanelt B, Brant SV, Steinauer ML, Maina GM, Kinuthia JM, Agola LE, Mwangi IN, Mungai BN, Mutuku MW, Mkoji GM, Loker ES (2009). *Schistosoma kisumuensis *n. sp. (Digenea: Schistosomatidae) from murid rodents in the Lake Victoria Basin, Kenya and its phylogenetic position among the *S. haematobium *group. Parasitology.

[B9] Steinauer ML, Hanelt Mwangi IN, Maina GM, Agola LE, Kinuthia JM, Mutuku MW, Mungai BN, Wilson WD, Mkoji GM, Loker ES (2008). Introgressive hybridisation of human and rodent schistosome parasites in western Kenya. Molecular Ecology.

